# Introducing Mechanically Assisted Cough for Patients With Progressive Neurological Disease: Patient–Physical Therapist Interaction and Physical Therapist Perspective

**DOI:** 10.1093/ptj/pzae012

**Published:** 2024-02-01

**Authors:** Anna Andersson-Watz, Malin Nygren-Bonnier, Elisabeth Bergdahl, Martin Eriksson Crommert, Mia Svantesson

**Affiliations:** Faculty of Medicine and Health, University Health Care Research Center, Örebro University, Örebro, Sweden; Medical Unit Occupational Therapy and Physiotherapy. Women’s Health and Allied Health Professionals Theme, Karolinska University Hospital, Huddinge, Sweden; Division of Physiotherapy, Department of Neurobiology, Care Sciences and Society, Karolinska Institute, Huddinge, Sweden; School of Health Sciences, Faculty of Medicine and Health, Örebro University, Örebro, Sweden; Faculty of Medicine and Health, University Health Care Research Center, Örebro University, Örebro, Sweden; Faculty of Medicine and Health, University Health Care Research Center, Örebro University, Örebro, Sweden

**Keywords:** Mechanical Insufflation–Exsufflation, Neuromuscular Disease, Physical Therapy, Qualitative

## Abstract

**Objective:**

The goal of this study was to explore the patient–physical therapist interaction and the physical therapist’s experience of the introductory session for mechanical insufflation–exsufflation (MI–E) device treatment for patients with progressive neurological disease.

**Methods:**

Qualitative content analysis of participant’s observation of interaction between patients and physical therapists during 9 MI–E introduction sessions in different clinical care settings and 10 follow-up interviews with 6 physical therapists.

**Results:**

The introduction of MI–E emerged as a process of instilling a sense of security in the patient. The process can be described in 4 steps: (1) gain understanding by being responsive to the person’s whole life situation; (2) share knowledge and expectations in a respectful and permissive way; (3) introduce the device in a gentle and reciprocal interactivity; and (4) adapt to home use in an inclusive dialog with the patient and their significant others. Physical therapists described a need for assurance to instill a sense of security in the patient, implying a need for confidence, competent peers, guiding yet flexible routines, and emotional support.

**Conclusion:**

Physical therapists have a need to foster assurance in employing a person-centered approach to make a patient feel secure in the process of introducing MI–E treatment. Multiple modes of professional knowledge were used together with action-based and relational-based ethics to facilitate a person-centered care approach. This seems to be a promising approach for providing good care when introducing MI–E to patients. Further research is needed to explore this from the patient’s perspective.

**Impact:**

This study added to the body of knowledge regarding MI–E treatment in relation to patients. This has direct implication, particularly for inexperienced physical therapists, for informed care for the patient during introduction. Our study also supports that person-centered care should be implemented at all levels of health care to make it possible for physical therapists to practice person-centered care.

## Introduction

Patients affected by progressive neurological disease in the later stages are prone to pulmonary complications,^1^ characterized by impaired ventilation and cough, which can increase morbidity and mortality.^2^ A common treatment method to support these patients is mechanically assisted cough, facilitated by a mechanical insufflation–exsufflation (MI–E) device. In many settings, physical therapists are responsible for introducing the MI–E treatment. This device compensates for weak breathing muscles by delivering a deep insufflation using positive air pressure followed immediately by negative air pressure, producing a forced exsufflation that clears the airway of mucus.^3–5^

There is no consensus in the literature regarding which settings for pressure, flow, and time are the most effective for treatment with MI–E. However, there are recommendations that the MI–E settings should be individually adapted to each patient.^6^ It has been shown that a successful introduction of MI–E, at least for home use, depends on the establishment of trust between the patient and the person who assists the patient, confidence in using the device, sufficient instructions, and practical training.^7^ Furthermore, the patient needs ongoing learning and follow-up.^8^

However, there is a lack of understanding about what kinds of interactions between the physical therapist and the patient work best to enable such individually adapted settings when introducing the MI–E device. In addition, the perspective of the physical therapist, who usually introduces the MI–E device to the patient, has not yet been described. Exploring these aspects can deepen our understanding of the introduction of MI–E for home use and can be useful when planning this treatment and introducing the device to the patient. Therefore, the aim was to explore the patient and physical therapist interaction during, as well as the physical therapist’s experience of, the introduction session for treatment with an MI–E device for patients with progressive neurological disease.

## Methods

A qualitative design was applied using participant’s observation and interviews.^9^^,^^10^

### Recruitment, Setting, and Sample

A consecutive sampling strategy was applied. Patients and physical therapists were recruited from 4 clinics in 3 different health care regions in the middle of Sweden from March 2018 to March 2019. First, patients diagnosed with progressive neurological disease, aged over 18 years, and scheduled for the introduction of cough-assist treatment for home use were invited to participate. Second, their physical therapists were invited. All patients (*n* = 11) and physical therapists (*n* = 6, 1 participating 3 times, 3 participating 2 times, and 2 participating 1 time) who were invited agreed to participate. The sample consisted of introduction sessions of MI–E (*n* = 9) and (*n* = 10) follow-up interviews with (*n* = 6) physical therapists (Table). One follow-up interview and 2 introduction sessions were missed out due to practical reasons.

**Table:** Characteristics of the Introduction Sessions and Participants^*a*^

**Characteristics**	**Value**
**Introduction sessions observed** (*n* = 9)	
Location University hospital County hospital	6 3
Session type Hospital inpatient Hospital outpatient Municipality home	1 5 3
Duration in min, mean (range)	51.7 (30–80)
**Patients** (*n* = 11)	
Sex Female Male	6 5
Age, y, mean (range)	60.6 (26–78)
Peak cough flow (liter/min), mean (range)	169.1 (0–280)
Diagnosis Amyotrophic lateral sclerosis Other progressive neurological diseases*^b^*	6 5
**Physical therapists** (*n* = 6)	
Role in session (*n* = 19) Observed Interviewed	9 10
Sex Female Male	5 1
Age, y, mean (range)	43.5 (34–63)
Experience as physical therapist, y, mean (range)	19.3 (11–36)
Experiences introducing MI–E for in-hospital use, y, mean (range)	8 (1–14)
Experience introducing MI–E for home use, y, mean (range)	5.2 (0–10)

^*a*^MI–E = mechanical insufflation–exsufflation.

^*b*^Multiple sclerosis, progressive supranuclear palsy, limb girdle muscular dystrophy, and fascia scapular humeral dystrophy.

### Data Collection

First, observations took place during the MI–E introduction sessions at either the patient’s municipality care home, in the hospital ward, or in a treatment room at the hospital. The observer (A.A.-W.) took field notes, guided by a study-specific observation protocol, inspired by Spradly.^11^ The main questions were: *what happens*, *what is said*, *and how is it said?* In the protocol, the time was noted at every 10th minute. The field notes were typed up and expanded, usually within 1 day.

Second, A.A.-W. conducted the follow-up semistructured interviews^12^ with the physical therapist introducing the device. Eight of these interviews took place at the interviewee’s workplace, and the remaining 2 were conducted by phone on the same day or within 3 days of the introduction session. The focus of each interview was on the therapist’s interactions with 1 individual patient. The interviews began with the main question: Can you please tell me about your experiences of carrying out the introduction with this individual patient? Follow-up questions, inspired by Price,^13^ consisted of “Please can you tell me more about …” or “Can you describe what you mean by …?” or as follow-up queries arising from the observations, such as “What was going through your mind when the patient sighed and looked down after trying the device for the third time?” Finally, general questions about the introduction from a wider perspective were posed, such as “Can you tell me your thoughts about how you follow-up with the patient about using the device? and How do you feel about encountering and instructing a patient with a severe chronic disease?” All of the interviews were audio-recorded and were transcribed verbatim.

### Data Analysis

We used qualitative content analysis with an inductive approach.^14^ The NVivo 13 software program, version 1.0 (released March 2020; QSR International Pty Ltd, Doncaster, Victoria, Australia), was used to facilitate the analysis (Suppl. Material 1). A.A.-W., a physical therapist and PhD student, was mainly responsible for collecting and analyzing the data. A.A.-W. has broad experience of neurological rehabilitation and of treatment with MI–E, mainly for inpatients. First, the data were read iteratively to make sense of the whole. Then, the text was divided into meaning units (parts sharing similar content) and was simultaneously formulated as condensed meaning units, with little or no degree of interpretation. Condensed sentences that were similar were given the same code. Then, a process of reformulating, merging, and splitting codes and simultaneously moving condensed meaning units was conducted in an iterative categorization process to build a categorization tree with abstraction to subcategories and categories. Interview data, comprising the physical therapists’ expressions about the specific sessions that were observed, were jointly analyzed. Interview data that generally related to the introduction from a wider perspective were analyzed separately. The abstraction phase continued while writing the results and during joint meetings with all of the coauthors, who were either physical therapists (M.E.C. and M.N.-B.) or registered nurses (E.B. and M.S.) with expertise in neurology, respiration, and qualitative methods until a main category emerged. Discussions and reflections from these meetings, and also peer review seminars at the research center, contributed to a broader, deeper, and more complex understanding of the phenomenon. The final analysis from participant’s observations and follow-up interviews emerged as a process (the main category) comprising a series of steps (the categories) (Suppl. Material 2). The final analysis of the physical therapists’ experiences emerged as categories and subcategories (Suppl. Material 3). The resulting process is exemplified in the results with composite descriptions,^9^ where different patient–physical therapist interactions from the field notes were merged into 1 story. This was used to provide a rich description of the patient–physical therapist interactions without identifying individual participants.

### Role of the Funding Source

The funders had no role in the study’s design, conduct, or reporting.

## Results

**Figure 1:**
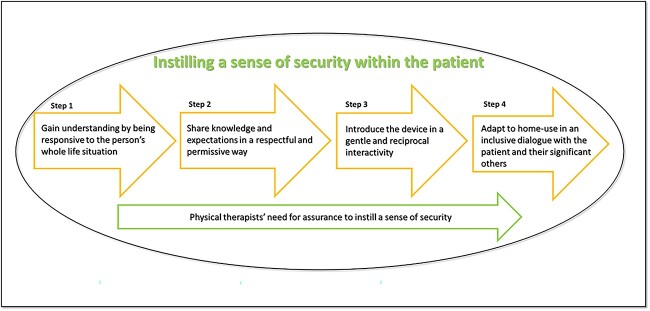
Illustration of the physical therapist instilling a sense of security within the patient during introduction of MI–E and their need for assurance to instill security to the patient. MI–E = mechanical insufflation–exsufflation.

The analysis of the observations and follow-up interviews revealed that the introduction of MI–E can be understood as a process of instilling a sense of security within the patient (Fig. 1). This process comprises 4 steps: gain understanding by being responsive to the person’s whole life situation, share knowledge and expectations about the device in a respectful and permissive way, introduce the device in a gentle and reciprocal interactivity, and adapt the device to home use in an inclusive dialog with the person and their significant others. To instill a sense of security within the patient, a need for assurance emerged. This was expressed as a need for competence, competent peers, guiding yet flexible routines, and emotional support (Fig. 1).

### Instilling a Sense of Security Within the Patient

#### Step 1: Gain Understanding by Being Responsive to the Person’s Whole Life Situation

**Figure 2:**
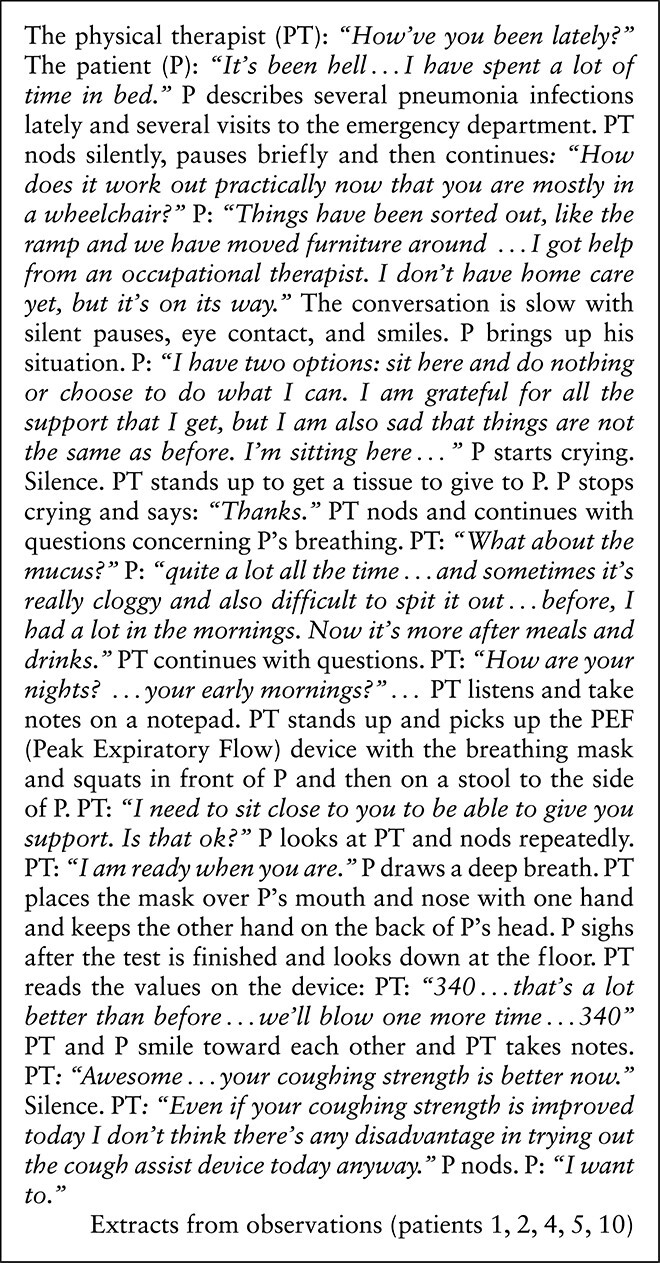
Step 1: gain understanding by being responsive to the person’s whole life situation. A composite description, drawing on observed interactions between physical therapists and patients in the introduction sessions.

During the introduction session with the patient, physical therapists tried to grasp the patient’s whole life situation by asking open-ended questions about the patient’s experiences of their life situation or breathing difficulties, or their expectations of the MI–E treatment. There was an attentiveness for both verbal and nonverbal reactions. Some patients showed emotional reactions and expressed difficulties about their situation (Fig. 2). Other patients provided brief responses and seemed more focused on moving forward. Paying attention to the patient’s reactions, asking follow-up questions, and making a clinical assessment seemed to provide both a specific understanding of the patient’s breathing problems as well as an understanding of the patient’s experiences of their whole life situation.

#### Step 2: Share Knowledge and Expectations in a Respectful and Permissive Way

**Figure 3:**
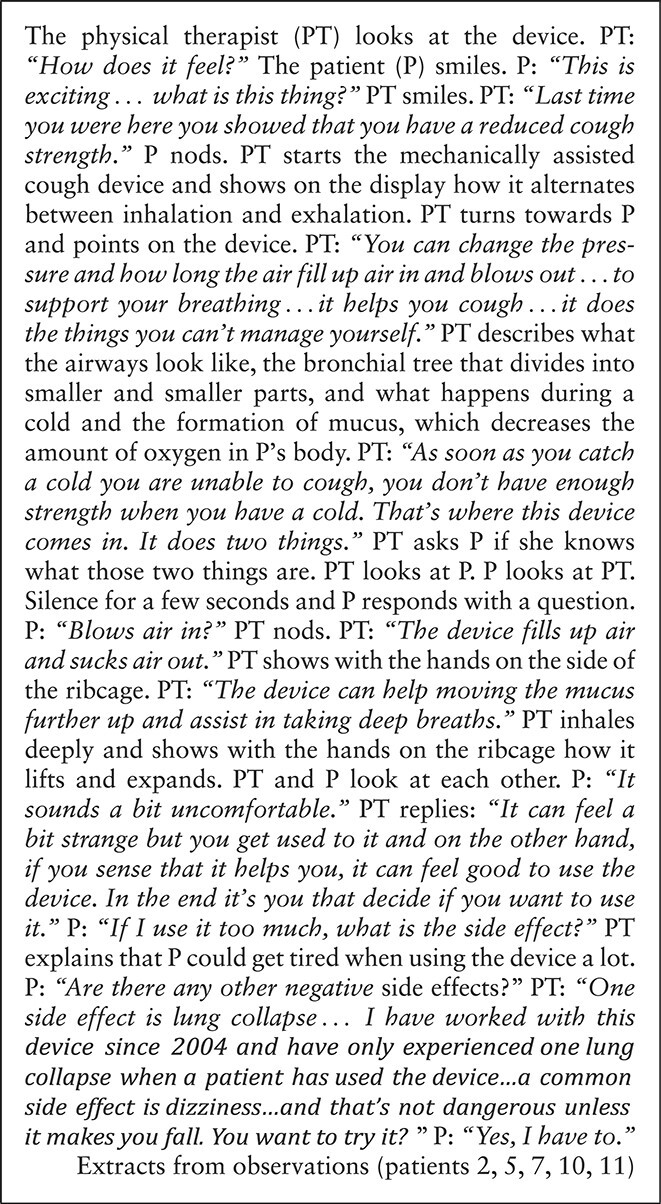
Step 2: share knowledge and expectations in a respectful and permissive way. A composite description, drawing on observed interactions between physical therapists and patients in the introduction sessions.

The physical therapists moved from establishing a broad understanding of the patient’s situation to narrowing their queries to focus on the patient’s preunderstanding of the MI–E treatment. Patients displayed reactions of curiosity, skepticism, or anxiety about whether the treatment would be unpleasant (Fig. 3). A joint sharing of knowledge took part, departing from discussing the patient’s illness experiences, with the patient reflecting and asking questions and the physical therapist listening, responding to difficulties, explaining, and clarifying (Fig. 3). It seemed as though this interaction infused not only a level of confidence for testing the treatment but also the confidence to refuse treatment. The physical therapists expressed respect and understanding of these responses. They described how they were trying to convey realistic expectations by explaining that the treatment will relieve the patient’s symptoms but not cure their illness.

#### Step 3: Introduce the Device in a Gentle and Reciprocal Interactivity

**Figure 4:**
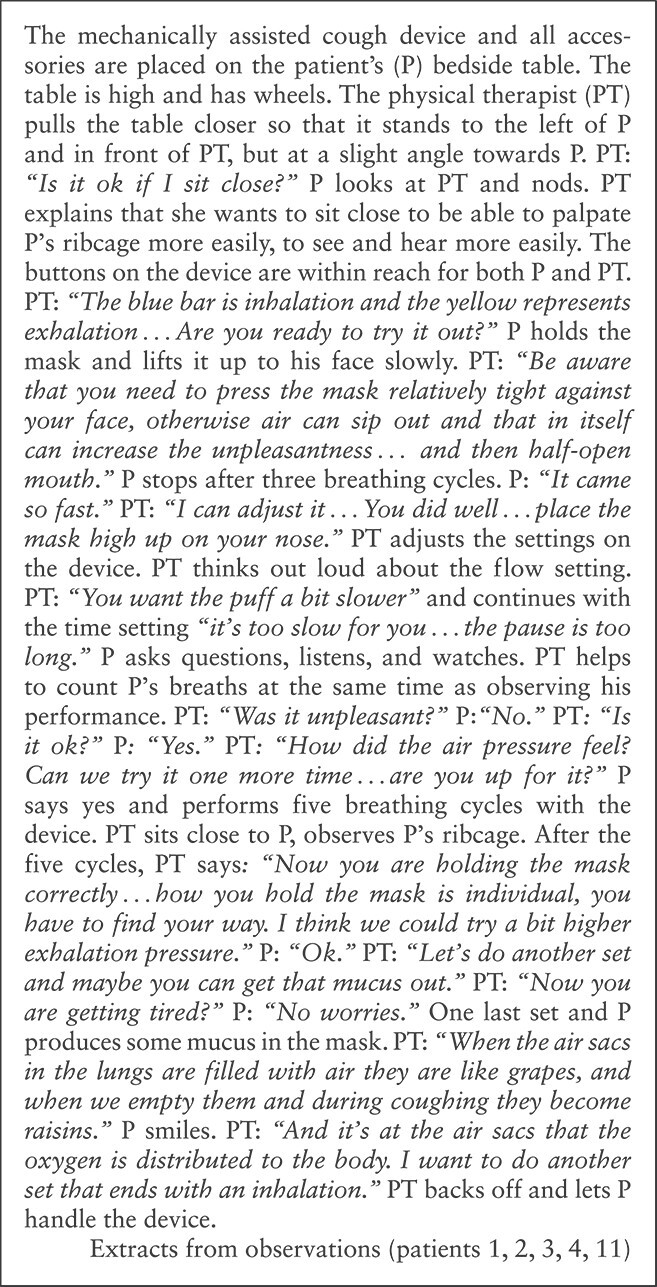
Step 3: introduce the device in a gentle and reciprocal interactivity. A composite description, drawing on observed interactions between physical therapists and patients in the introduction sessions.

Introducing the device by starting with a low-pressure setting seemed to dominate, with 10 to 15 cm H2O corresponding to a normal breath or a sigh. The initial pressure could also be matched with the patient’s prescribed home ventilator settings. For time settings, some physical therapists used a manual setting, where they followed the patient’s breathing pattern to manually control the timing of the insufflation and exsufflation on the device. Because some physical therapists expressed that they knew that exsufflation could be uncomfortable for the patient, they started with the insufflation, adjusting it as necessary, and postponed the introduction of the exsufflation phase. For the same reason, when they began to introduce the exsufflation phase, the time intervals between breaths were set shorter, and the intervals were adjusted according to the patient’s needs and comfort. Others started by using the automatic mode, where the time intervals were preset for the whole breathing cycle, guided by the clinical assessment of the patient’s breathing patterns. Regardless of the initial approach, the physical therapists optimized time, flow, and pressure stepwise, together with being attentive to each patient’s individual reactions (Fig. 4).

Adjusting the settings also served as an opportunity for the patients to learn to operate the device. In this learning process, the physical therapists guided the patient both verbally and physically (Fig. 4). For example, the numbers displayed on the device demonstrated to the patient how their technique had improved with practice (Fig. 4). Learning how to adjust the settings and use the device was observed to be achieved in 1 or several visits.

#### Step 4: Adapt to Home Use in an Inclusive Dialog With the Patient and Their Significant Others

**Figure 5:**
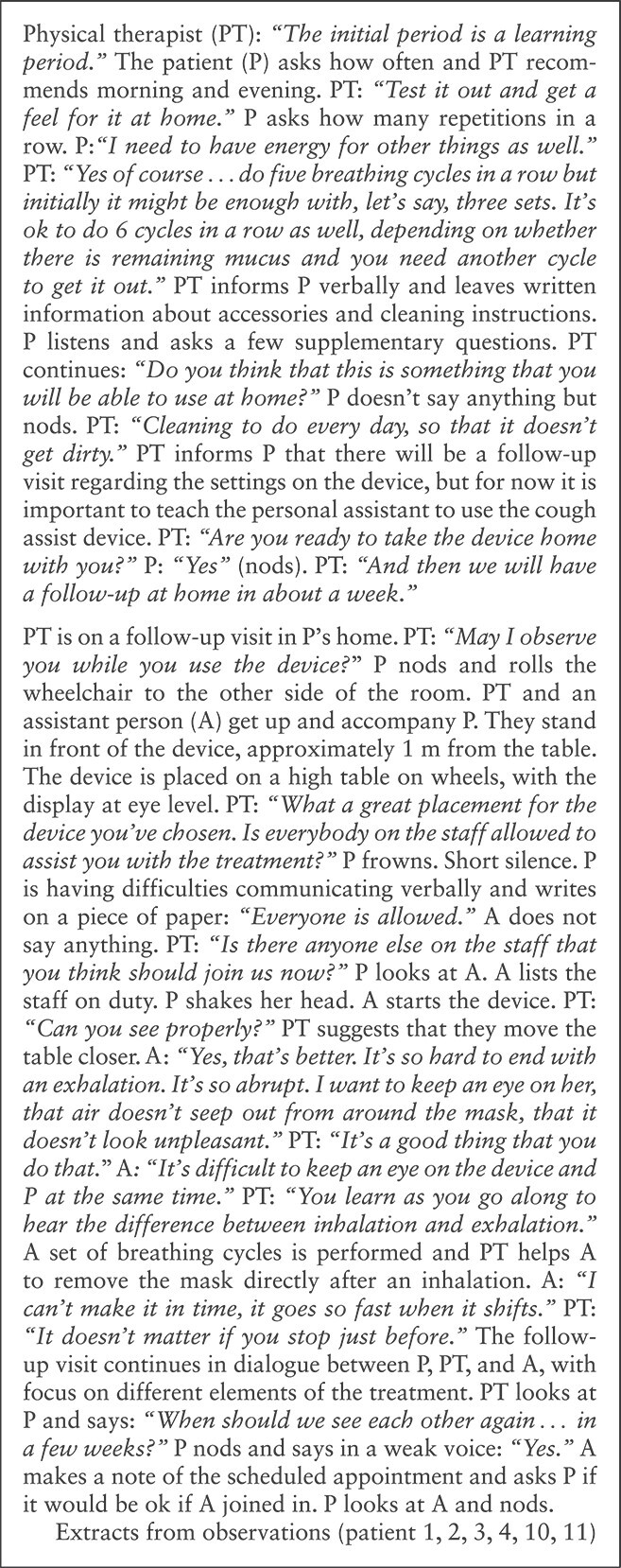
Step 4: adapt to home use in an inclusive dialog with the patient and their significant others. Two composite descriptions, drawing on observed interactions between physical therapists, patients, and assistant person in the introduction sessions.

Patients’ dependency on physical support from personal assistants, assistant nurses, or family caregivers to handle the device and its components dominated. In an open dialog, the physical therapists identified those aspects of the MI–E treatment in which the patient might need support when using the device at home. The physical therapists explained that, to empower the patient in this situation, they involved the patient in the introduction and gave the patient a feeling that this is something “I can do and I know what it is.” However, transferring this feeling of control from the clinical setting to the home care setting could be challenging. Some patients expressed certainty and self-confidence in home use, while others were uncertain about their own ability, and that of their significant others, to cope with the device at home. Patients expressed having negative experiences in similar situations, for example, in not being provided with sufficient support in home-exercise programs. The physical therapists responded by taking responsibility for relaying information about the patient’s needs to other health care professionals involved in their care. During the introduction session, patients either attended with or without a supportive person. When supportive persons participated, they received the same information as the patients and were instructed to use the device together with the patients. Some patients expressed that they alone took responsibility for teaching the supportive persons at home, while others asked for help to instruct them (Fig. 5). The physical therapists felt that, when they were unsure about the level of support available in the person’s home environment, introducing the MI–E device in the patient’s home was valuable. Home visits were then facilitated to adapt the support to meet the individual and to integrate the treatment into the home environment as well as to facilitate a collaboration with other health care professionals involved in the care (Fig. 5). Unfortunately, not all physical therapists were able to perform home visits.

To learn how to use the device, the physical therapists recommended that the patients use it at home daily. The target was 5 breathing cycles in a row, each consisting of 5 inhalations and exhalations, but this could be adapted to whatever the individual patient could manage comfortably. These recommendations could also be adjusted according to the patient’s individual breathing difficulties and the need for mobilization of mucus (Fig. 5). In an encouraging dialog with the patient, the physical therapists made it clear that, ultimately, it was always the patient’s own choice that would govern their use of MI–E (Fig. 5).

### Physical Therapists’ Need for Assurance

A need for assurance in introducing MI–E emerged through the physical therapists’ need for reliance on their own competence, competent peers, guiding yet flexible routines, and emotional support.

### Need for Competence

Knowledge of the treatment and familiarity with the device was perceived as being essential when conducting the introduction. This entailed using it themselves and getting hands-on experience. Knowledge about the different functions of the device was described as providing confidence in teaching other persons. Furthermore, medical knowledge, such as disease progression, comorbidities, symptoms, and the physiological effects of the treatment, was expressed as providing important information to safely optimize the settings of the MI–E device. The physical therapists described how making small adjustments could make a difference for the patient’s comfort. A form of reasoning emerged as they weighed the implications of the patient’s illness symptoms and disease against what could be gained for the patient by accepting the treatment.

He quit smoking four months ago and after that, he had problems, so I was concerned whether this [MI-E] was appropriate. Nevertheless, he had some issues with mucus, so I concluded that it was good that he still could try … many with COPD have a risk, they are frailer than others … simply greater risk … but he had three pneumonias in four months and it was pretty tough with the mucus … so I thought that he probably need a cough assist [MI-E] device, it is probably a greater risk not having it, than having it. (Physical therapist E)

### Need for Competent Peers

The physical therapists described having a need for peer support, with interdisciplinary input from other health care professionals such as nurses, doctors, speech therapists, and occupational therapists, as being important for addressing the patient’s whole-care needs. This eased the introduction process for the physical therapist by providing more time to focus on the introduction of MI–E, while at the same time being reassured that the patient’s other needs were also being addressed, such as measures to compensate for swallowing difficulties or aids to increase independence in their daily activities. When feelings of inadequacy in conducting the introduction surfaced, the physical therapists described how being able to rely on the knowledge and support of a close peer was invaluable.

I had one that said: … you can call when you want to, if there is anything I can always come to assist … it is a comfort for me, which in turn is a comfort for the patient. (Physical therapist F)

By contrast, the absence of peer support felt just as problematic in relation to unanswered questions regarding the patient’s care or delayed care. Having a knowledgeable physician to back them up was experienced as being safe and preferable to feeling unsafe with a physician who lacked specific knowledge and was unfamiliar with the patient. They also described experiences of preferring to take their own control of prescribing the MI–E settings.

I’ve received a referral that states to try 5–10 cm water … It’s completely unreasonable. It has to be somebody who is familiar with the patient or can get information about the patient and someone who knows a little about cough assist [MI-E] treatment. (Physical therapist C)

### Need for Guiding Yet Flexible Routines

Written instructions for remembering the many elements in the introduction process were valued, but they were expressed as only being useful if they allowed for making adaptations for the patient’s specific needs and ability to participate. Variation from the instructions could imply dividing the introduction into several short sessions and/or moving the introduction session to take place in the patient’s home in order to avoid an exhausting journey to the hospital. Physical therapists felt encouraged when their managers trusted their professional judgment and offered flexible routines and work schedules, enabling them to adapt the introduction to the patient’s needs. They also experienced that making such time-consuming individual adaptations was challenging, given their already high workload. Here, they felt forced to prioritize some patients over others or provide the introductions outside working hours.

We have limited resources, we have limited time. If I go out [on a home visit] where should I take that time from? I scheduled it right before lunch this time because I knew that if it dragged on, then it’s only my lunch break that gets affected, really, because I thought that was easier to fix, than late in the afternoon... (Physical therapist B)

### Need for Emotional Support

A need for support to deal with one’s emotions was expressed. Struggling with feelings of having a bad conscience related to the guilt of not being sufficiently responsive to the patient’s burden of illness was conveyed. One worry was whether one’s own feelings would have a negative impact on the meeting with the next patient, perhaps trivializing a less severely ill patient’s issues.

One way to manage these difficult feelings could be to focus on positive elements of care by being responsive and helpful. Sharing one’s feelings with a physical therapist colleague was also expressed as being supportive. Furthermore, reflecting together in interprofessional teams within an open climate was experienced as being valuable for managing emotional reactions. There was also an expressed need for professional psychological support, particularly in periods where many severely ill patients were treated. This need also seemed to include reflecting ethically about difficult patient encounters, but this was difficult to accomplish.

… it’s tough emotionally, we don’t have anything concrete. We’ve talked about whether we could have some form of coaching to support us. We have access to a counselor and a hospital chaplain, but that’s mostly for the patients, it’s not for us. (Physical therapist D)

## Discussion

The significance of the value of security emerged in our analyses of the introduction sessions for treatment with MI–E for patients diagnosed with progressive neurological disease. The ability to instill a sense of security within the patient addressed the physical therapists’ need to provide assurance (Fig. 1). We observed security as being a central value. The sense of security was significant, in particular for this patient group, because it seems to render a sense of control.^15^ One might even claim that security should be recognized as an ethical value, that is, as an intrinsic value in itself and not as a means to facilitate something else.^16^ From our results, a sense of security seemed to be instilled in the patient through an interaction of embodied responsiveness, respectfulness, permissiveness, gentleness, and inclusiveness. We consider all these characteristics to be virtues of relational ethics. The importance of security observed here aligns with studies involving other patient groups, such as those in intensive care, palliative care, and older people care.^15–17^ In an older people care context, a sense of security was described by both health care professionals and patients as being an internal sense of faith and trust in oneself and others.^16^ Having a sense of security is vitally important for patients who have a limited life expectancy. This because a low sense of security is associated with higher symptom intensity and lower quality of life.^17^ According to Milberg et al,^17^ infusing a sense of security supports the patient’s sense of mastery, their identity, and their perception of a secure interaction and helps health care professionals to attune to the family members’ situation.^17^

A palliative care approach goes hand in hand with the philosophy of person-centered care,^18^^,^^19^ an approach that the physical therapists in this study practiced implicitly. According to Ekman et al, person-centered care implies initiating a partnership by combining the patient’s narrative^20^ with responsive interaction based on action-based ethics and relational ethics.^21^ Furthermore, the process implied the cocreation of shared experiences and learning from each other^22^ along with an individually adapted approach to promoting patient-tailored education and communication.^23^

The concept of the patient’s narrative^20–22^ is implied in our study by the physical therapists departing from the patient’s own descriptions about their symptoms, illness, and situation. This has been reported to contribute insight into the personal meaning of illness^21^ and improve the prerequisites for establishing person-centered care planning.^24^ Breathing is vital, and, when impeded, essential physiology is affected in fundamental ways. Observing the interactions in our study, there seemed to be the embodiment of responsive understanding of the significance of breathing difficulties for the patient. This understanding corresponds with a person-centered palliative care philosophy that focuses on the quality of life and includes an individual and holistic approach to caring for the patient.^18^^,^^19^ Responsive interaction implies being responsive to each patient’s individual needs and includes asking questions and listening sincerely to experiences, which are also key elements of promoting a positive therapeutic alliance^22^ in person-centered care.^20^ The interactions observed in our study provide exemplars of the characteristics of a good clinical physical therapist, which include: “the ability to intertwine technical competence with a relational way of being, described as responsive, ethical, communicative, caring and collaborative.”^25^

Acknowledging patients’ vulnerability and their inferior position of authority in relation to the health care professional is crucial.^21^ Here, seeing the patient as a person is paramount; it promotes a shift away from seeing the patient as a passive target to acknowledging them as an active partner in planning and carrying out their care.^21^ Ekman^21^ describes person-centered care as a process of building a relationship—one that requires the employing of “ethics as a springboard” ^21^ as a starting point for establishing a respectful and mutual alliance or partnership. Ekman^21^ draws on Ricoeur’s notion that ethics as a goal to achieving a good life should supersede any moral considerations. When met with moral dilemmas, health care professionals can draw on their ethical aim—to promote health and well-being—in resolving them. This aligns with our observations of how the physical therapists addressed their perplexities in feeling inadequate. Person-centered care integrates both professional knowledge and practical wisdom,^21^ a combination which, according to Aristotle, must include ethics. This suggests that person-centered care comprises both action-oriented ethics, that is, tailoring care measures for the patient’s well-being,^21^ and relational-oriented ethics, capturing the needs of the patient in responsive interaction.^26^ For us, building such a relationship therefore implies the enrolment of relational virtues in the interaction (as listed above).

Similarly, cocreation in the interaction of sharing experiences and learning from each other^22^ implied sharing knowledge and respecting the patient’s decision about how often they would use the device, and forming an understanding of the home environment to facilitate treatment in the personal space. Cocreation as a phenomenon is described within palliative home health care as multiple parallel processes, which, in being unique to each individual patient, can be useful to facilitate patient care goals.^27^

Facilitating an individually adapted approach, as described in our study, aligns with the results of previous research.^6^ Individuality has previously been defined as “patient-tailored education, communication, and treatment”,^28^ and encompasses, as Ekman^21^ states, “care actions tailored to each patient’s wellbeing.” ^21^ Such tailoring was evident in the present introduction process. Instructions and guidance about the device as a treatment were guided by gaining an insight into the patient’s preunderstanding, which was further facilitated by a mutual sharing and cocreation of knowledge. Moreover, being attentive to both verbal and nonverbal communication, and attuning to both physical and psychological reactions when instructing how to set and use the device, seemed to be important for tailoring treatment. This finding aligns with previous research that describes how recognizing and being familiar with objects and situations promote and strengthen a sense of security.^17^ This aspect of our results, where the patient learns from both the physical and verbal guidance provided by the physical therapist, is also a good example of experiential learning, or *“learning by doing*,” which was first proposed by the philosopher and educationist John Dewey.^29^ In our study, home visits were found to be particularly valuable for adapting individual tailored support, integrating the treatment into the home environment, and facilitating collaboration with other health care staff.

Enrolling person-centered care appears to be a promising approach for facilitating the introduction of MI–E for patients with progressive neurological disease and impaired cough. Furthermore, enrolling this approach also promotes the further development of care for this patient group. Although patients receiving physical therapy have expressed a desire for person-centered care,^23^ earlier research indicates that physical therapists seem to find it difficult to incorporate this approach into their practices.^30^^,^^31^ While the challenges of implementing person-centered care were not the focus of this study, our findings seem to contradict this, as all the physical therapists were observed to consistently integrate this ethos in their professional practices. Elsewhere, it has been suggested that the person-centered concept needs to move from person-centered moments to person-centered care^32^ and embed all levels of the health care system from government policy to delivery of care by individual clinicians.^33^ Our findings appear to support this move.

The significance of needing to instill the patient with a sense of security also implied a need for the physical therapist’s own competence, competent peers, guiding yet flexible routines, and emotional support. From a health care professional’s perspective, a sense of security, together with a system that facilitates trustworthy and reliable relationships over time, has been suggested to be important for providing good care.^15^ Building a partnership with the patient is a core aspect of the person-centered care approach, and it is not only limited to involving the patient but also involves partnership among team members around the patient to provide good care.^34^ In a health care context, as revealed in our study, this partnership between colleagues seems to be important for meeting the need for assurance in their own competence and for receiving emotional support. Furthermore, a need for guiding yet flexible routines is in line with earlier research describing prerequisites for instilling security.^16^ Person-centered care implies an ongoing process, one that is derived from the additional element of considering patient-goals and underpinned by an ethical approach rather than from simply following standardized guidelines.^34^

In the process of conducting the MI–E introduction, the physical therapists’ professional reasoning was mediated by their knowledge of the device and their knowledge of the patient’s illness as well as the patient’s verbal responses and body language. The physical therapists aimed to prescribe higher exsufflation pressures to produce a cough effective enough to mobilize mucus,^35^ but at the same time, were aware that these high pressures could cause airway compression and therefore be counterproductive.^36^ This illustrates how the interaction between the physical therapist and patient involves a complex process of assembling multiple elements of professional and technical knowledge to provide the best possible care for the patient.

Alongside the need for access to more hands-on support from peers, a need for emotional support was also expressed. This could take the form of professional psychological support and also signified a need for reflecting together with professional peers. In fact, a kind of moral distress, such as laying the burden of providing patient-centered care solely on one’s own shoulders and blaming oneself for emotional responses, was implicitly conveyed. This encompassed feelings of having a bad conscience relating to the guilt of not having enough time to sufficiently address the patients’ suffering. Such moral distress has also been experienced by frontline COVID staff.^37^ As Ekman states, person-centered care relies on using “ethics as a springboard”^21^ in the guidance of health care professionals. However, as our study suggests, it is essential that these reflections on ethics are revisited and continue to inform professionals throughout the entire care process. Giving clinicians opportunities to reflect in a structured way about what constitutes good care, such as those provided via moral case deliberation, can make these ethical issues visible and enable moral learning.^38^^,^^39^

### Methodological Considerations

A major strength of this study is that it allowed us to gain realistic insights into a specific phenomenon through the combination of performing participant’s observations and interviews.^9^^,^^10^ This increased the richness of the data and the trustworthiness of the findings, as the field notes from the observed interactions in the clinical encounters were used to refine the questions posed to the physical therapists in the follow-up interviews. Thus, we were able to focus more deeply on the therapists’ approach to guiding the interactions with the patients. This study was based on 9 sessions of observing patient–physical therapist interactions while introducing MI–E, which could be viewed as a somewhat narrow sample and thereby a limitation to the study. However, our sample comprised a mix of settings for delivering the introduction and variation in terms of patient diagnosis, gender, and age. We think that this variation helps to increase the trustworthiness of the study. A potential weakness regarding sampling was delegating the recruitment of patients to the physical therapists while they were working in the clinic. They reported that this was sometimes difficult to manage. Another potential weakness in terms of trustworthiness and transferability might be that the researcher observing the MI–E introduction sessions and performing the interviews may have influenced the participants. To minimize this risk, the researcher contacted the patients and the physical therapists before the observations and interviews took place to clarify that the aim of the study was not to judge or evaluate their performance and was only to describe the interaction with as little interference as possible. Time was also set aside to allow the patients and physical therapists to ask questions about the purpose of the study and their involvement in it. During the introduction session, the researcher tried to keep a low profile when observing, for example, by not interrupting the encounter by asking questions.

## Conclusion

In our study, the physical therapists implicitly employed a person-centered approach to make the patient feel secure in the process of introducing MI–E treatment. A need to foster assurance emerged as being important to instill a sense of security within the patient. Physical therapists used multiple modes of professional knowledge together with action-based ethics as well relational ethics. A person-centered care approach, facilitated by these 2 areas of ethics, seems to be a promising for providing good care when introducing MI–E for the patient. Moreover, this knowledge contributes more broadly to an understanding of a relational-oriented ethics foundation for person-centered care. Further research is needed to explore the patients’ experience of living with a progressive neurological disease, their experience of the introduction of MI–E, and of the home-treatment with the device. In a clinical context, that knowledge, in addition to the findings in the present study, could be used to provide a good care for these patients.

## Supplementary Material

PTJ-2022-0665_R3_Supplementary_Material_1_pzae012

PTJ-2022-0665_R3_Supplementary_Material_2_au_pzae012

PTJ-2022-0665_R3_Supplementary_Material_3_pzae012
