# Supplementary material for: Introducing Mechanically Assisted Cough for Patients With Progressive Neurological Disease: Patient–Physical Therapist Interaction and Physical Therapist Perspective
Source: Phys Ther. 2024 Feb 1;104(5):pzae012. doi: 10.1093/ptj/pzae012 (PMC11140267; doi:10.1093/ptj/pzae012)

Supplementary Material 1 – Screenshot from the software analysis program NVivo showing the analysis steps from the category *Introduce the device in a gentle and reciprocal interactivity*

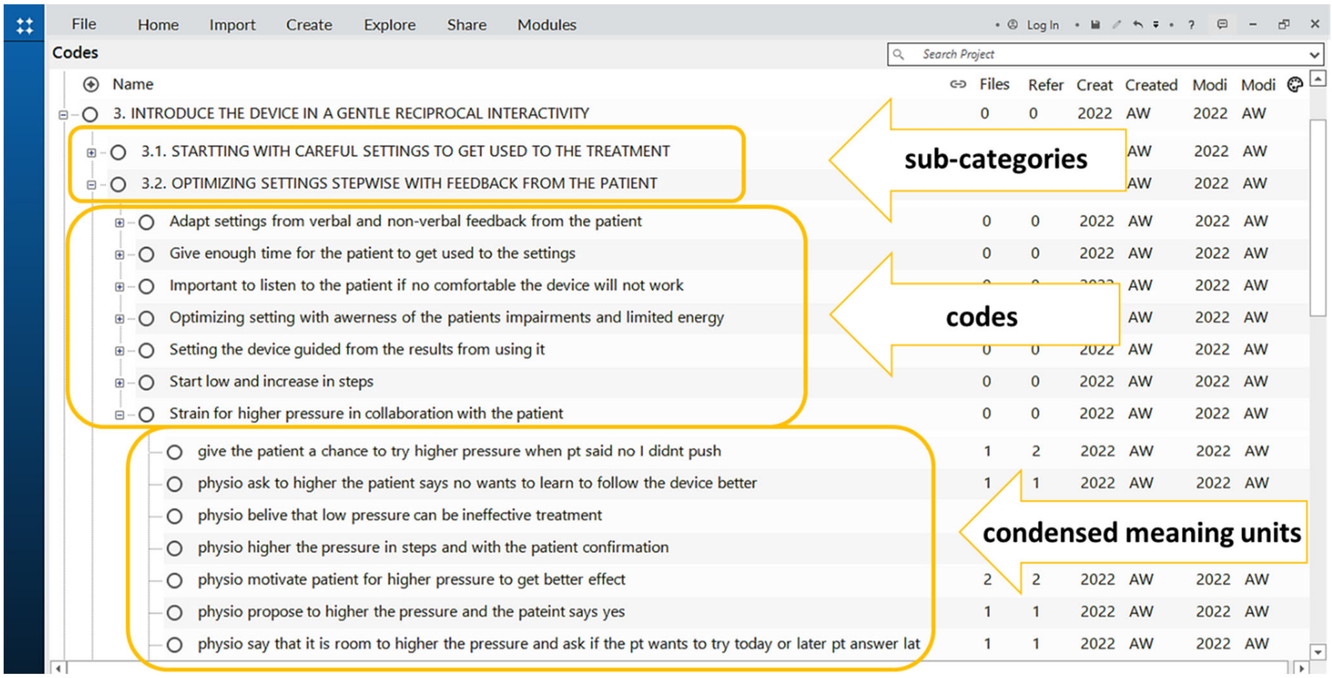

Supplement: PTJ-2022-0665_R3_Supplementary_Material_1_pzae012 [file ptj-2022-0665_r3_supplementary_material_1_pzae012.pdf]
