# Supplementary material for: Introducing Mechanically Assisted Cough for Patients With Progressive Neurological Disease: Patient–Physical Therapist Interaction and Physical Therapist Perspective
Source: Phys Ther. 2024 Feb 1;104(5):pzae012. doi: 10.1093/ptj/pzae012 (PMC11140267; doi:10.1093/ptj/pzae012)
Supplement: PTJ-2022-0665_R3_Supplementary_Material_2_au_pzae012 [file ptj-2022-0665_r3_supplementary_material_2_au_pzae012.pdf]

Supplementary Material 2 – Results of the qualitative content analysis, from participant-observations of introduction-sessions of the MI-E device and follow-up interviews with the physical therapists introducing the device, divided into SUBCATEGORIES, CATEGORIES, and MAIN CATEGORY.

SUBCATEGORIES	CATEGORIES	MAIN CATEGORY
• Considering the patient's whole life situation by open-ended questions<br>• Connecting with patients by attentiveness towards their narrations<br>• Picturing the patient's breathing by directed questions and clinical assessment	Gain understanding by being responsive to the person's whole life-situation	Instilling a sense of security within the patient
• Engaging in dialogue for the patient's preunderstanding of the treatment<br>• Clarifying eventual preventive and palliative outcomes of the treatment, guided by the patient's illness experiences<br>• Presenting opportunities in the situation and respecting the patient's choices	Share knowledge and expectations in a respectful and permissive way	
• Starting with low settings to physically and mentally adapt to the treatment<br>• Optimizing the settings stepwise with attention for the patients reactions<br>• Teaching the patient to handle the device through individually guided use	Introduce the device in a gentle and reciprocal interactivity	
• Recommended use departing from the patient's needs and wishes<br>• Becoming familiar with the patient's home environment to enable need based adaptations<br>• Adapting guidance to personal assistants based on the patient's support needs	Adapt to home-use in an inclusive dialogue with the patient and their significant others	
