# Supplementary material for: Introducing Mechanically Assisted Cough for Patients With Progressive Neurological Disease: Patient–Physical Therapist Interaction and Physical Therapist Perspective
Source: Phys Ther. 2024 Feb 1;104(5):pzae012. doi: 10.1093/ptj/pzae012 (PMC11140267; doi:10.1093/ptj/pzae012)
Supplement: PTJ-2022-0665_R3_Supplementary_Material_3_pzae012 [file ptj-2022-0665_r3_supplementary_material_3_pzae012.pdf]

Supplementary Material 3 - Results of the qualitative content analysis, from follow-up interviews with the physical therapists introducing the device, divided into SUBCATEGORIES and CATEGORIES

**SUBCATEGORIES**	**CATEGORY**
• Need for competence<br>• Need for competent peers<br>• Need for guiding yet flexible routines<br>• Need for emotional support	Physical therapists' need for assurance
